# Functional Recovery After 18 Sessions of Radio Electric Asymmetric Conveyor Tissue Optimization Reparative Protocol for Hill-Sachs Lesion in a Post-traumatic Shoulder Dislocation

**DOI:** 10.7759/cureus.78495

**Published:** 2025-02-04

**Authors:** Alessandro Castagna, Enrico Castagna, Vania Fontani, Salvatore Rinaldi

**Affiliations:** 1 Department of Research, Rinaldi Fontani Foundation, Florence, ITA; 2 School of Specialization in Physical and Rehabilitation Medicine, Sapienza University of Rome, Rome, ITA; 3 Department of Regenerative Medicine, Rinaldi Fontani Institute, Florence, ITA

**Keywords:** functional motor recovery, hill-sachs lesion, radio electric asymmetric conveyor, reparative medicine, shoulder anterior dislocation

## Abstract

This case report describes the clinical progress of a 22-year-old male patient diagnosed with a consolidating Hill-Sachs lesion, treated with 18 sessions of Tissue Optimization Reparative (TO-Rpr) protocol, specific to Radio Electric Asymmetric Conveyor (REAC) technology. At baseline, the patient reported persistent pain with a numeric rating scale (NRS) score of 7 out of 10 and significant functional limitations of the shoulder (flexion 90°, abduction 70°, external rotation 20°), accompanied by bone marrow edema, tendinous alterations, and significant inflammation. The therapeutic protocol aimed to modulate tissue bioelectric activity to promote reparative processes and reduce inflammation. Follow-up MRI, performed four months after treatment, revealed complete resolution of bone marrow edema, normalization of tendinous structures, and reduced cortical irregularities. Clinically, flexion improved to 160°, abduction to 150°, and external rotation to 70°, with muscle strength restored to 5/5 and pain reduced to 0/10 on the NRS. This case highlights the role of REAC TO-Rpr treatment in managing complex joint lesions, showcasing its potential to significantly improve clinical and radiological parameters within a short timeframe.

## Introduction

Hill-Sachs lesions are among the most common consequences of anterior glenohumeral dislocations, characterized by compression fractures on the posterolateral surface of the humeral head [1,2]. These injuries are particularly prevalent among younger, active individuals engaged in sports or physical activities, where shoulder stability is critical for performance and quality of life. Such lesions frequently lead to pain, restricted range of motion, biomechanical imbalances, and joint instability. These functional impairments significantly impact the quality of life, affecting the ability to perform daily and sports activities [3]. Furthermore, untreated or poorly managed cases often result in recurrent dislocations and chronic instability, necessitating more invasive interventions.

Conventional approaches to Hill-Sachs lesions typically include conservative treatments, such as physical therapy and pharmacological pain management [4], as well as surgical techniques like remplissage and bone grafting [5]. However, these methods have limitations, particularly in addressing chronic inflammation and structural abnormalities. The efficacy of conservative management may be hindered by persistent inflammatory processes and biochemical alterations within the affected tissues [6]. Surgical options, while effective in stabilizing the joint, are associated with significant risks, extended recovery periods, and variable outcomes [7].

Recent advancements in reparative and regenerative medicine have introduced innovative treatment modalities aimed at addressing the underlying pathophysiological mechanisms of such injuries. Among these, the Radio Electric Asymmetric Conveyor (REAC) technology represents a promising non-invasive solution. The Tissue Optimization Reparative (TO-Rpr) protocol [8-10], a specific application of REAC technology, offers several unique advantages over traditional non-invasive or surgical approaches. It does not rely on invasive procedures or pharmacological agents, ensuring a safer and more patient-centric treatment. Additionally, the protocol’s standardized parameters reduce operator-dependent variability, promoting reproducibility and consistent therapeutic outcomes. The TO-Rpr protocol utilizes asymmetrically conveyed weak radioelectric fields to modulate cellular bioelectric activity [11]. This modulation promotes reparative processes, reduces inflammation, and facilitates functional recovery in a wide range of musculoskeletal conditions.

This case report presents the application of the TO-Rpr protocol in the management of a Hill-Sachs lesion, highlighting the significant clinical and radiological improvements observed. By addressing both the symptomatic and structural aspects of the injury, this approach underscores the potential of REAC technology in enhancing outcomes for complex joint pathologies.

## Case presentation

A 22-year-old male patient with no significant medical history presented with persistent left shoulder pain and severely limited mobility following trauma incurred during a waterpark slide accident. The traumatic event resulted in an anterior shoulder dislocation, which was promptly managed with an emergency reduction. After the initial intervention, the patient was advised to immobilize the shoulder using a brace for two weeks, followed by a prescribed rehabilitation program. Despite compliance with the plan, which included targeted physical therapy and exercises, the patient experienced no meaningful improvement in pain levels, recorded at 7/10 on the numeric rating scale (NRS), or in functional mobility.

The injury significantly affected the patient’s psychological well-being and daily life. He reported frustration and emotional distress due to his inability to engage in sports and perform essential tasks such as lifting objects or even dressing without assistance. These limitations also impacted his professional and social activities, further exacerbating his emotional challenges. He expressed concern about the potential for chronic instability and long-term disability.

The patient denied any prior injuries or pre-existing conditions that might have influenced his recovery. His medical history was unremarkable, and there were no indications of comorbidities that could impede the effectiveness of the rehabilitation process or the TO-Rpr treatment. Due to the lack of progress with conventional rehabilitation methods and the ongoing functional and psychological challenges, the decision was made to proceed with the TO-Rpr protocol as an alternative therapeutic approach.

Diagnostic methods

**Figure 1:**
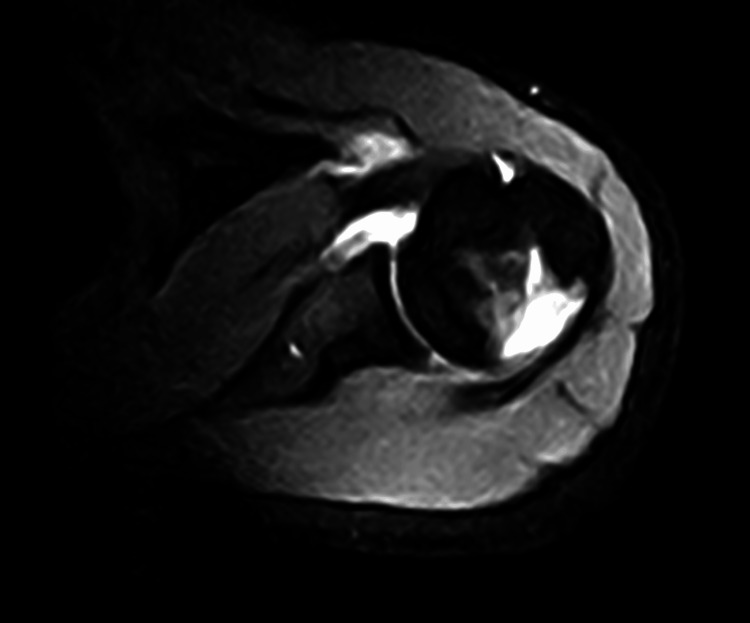
Pretreatment magnetic resonance imaging (MRI) of the shoulder. The initial scan reveals a consolidating Hill-Sachs lesion on the posterolateral surface of the humeral head, indicative of structural damage and associated inflammation.

Three weeks post-trauma, magnetic resonance imaging (MRI) was performed to evaluate the extent of the structural damage and associated inflammation. The imaging revealed a consolidating Hill-Sachs lesion on the posterolateral surface of the humeral head (Figure 1).

Bone marrow edema was observed extending into the proximal humeral diaphysis, alongside notable subscapular and infraspinatus tendinosis, indicative of elongation and overuse. The coracobrachialis muscle exhibited edema, and bursitis was evident in the subscapular region. Tenosynovitis of the long head of the biceps tendon was also present, accompanied by fluid accumulation in the scapulohumeral joint and widening of the inferior axillary recess, indicative of active inflammation.

The severity of the lesion was assessed based on the extent of cortical irregularities, the presence of bone marrow edema, and the associated soft tissue involvement. While formal grading criteria for Hill-Sachs lesions were not explicitly applied in this case, the imaging findings indicated a significant impact on the biomechanical integrity of the shoulder joint. This comprehensive assessment highlighted the need for an intervention capable of addressing both structural damage and chronic inflammation.

The imaging results were pivotal in guiding the decision to use the TO-Rpr protocol. The observed bone marrow edema and soft tissue inflammation suggested that a treatment targeting cellular bioelectric modulation could facilitate reparative processes and reduce the underlying inflammation. Furthermore, the absence of contraindications to the TO-Rpr protocol and the patient’s limited response to conventional rehabilitation reinforced its suitability as an alternative therapeutic option.

Therapeutic intervention

The patient underwent a complete cycle of TO-Rpr treatment using the REAC BENE 110 device (ASMED, Scandicci, Italy). The protocol was specifically chosen for this case due to its non-invasive nature, its demonstrated efficacy in modulating bioelectric activity to promote tissue repair and reduce inflammation, and its suitability for the lesion type. The chronic inflammation, extensive bone marrow edema, and soft tissue involvement observed on imaging indicated that a therapeutic approach capable of addressing both biochemical and biomechanical dysfunctions was required. The patient's failure to respond to conventional rehabilitation methods and his preference for avoiding surgical interventions further reinforced the decision to proceed with TO-Rpr treatment.

The treatment protocol consisted of 18 sessions administered over five consecutive days. Each session, lasting 15 minutes, involved the placement of the asymmetric conveyor probe (ACP) directly on the affected shoulder. The pre-set parameters of the TO-Rpr protocol ensured precise, reproducible bioelectric modulation, targeting cellular reparative mechanisms and reducing inflammation. Treatment sessions were conducted at intervals of no less than one hour and no more than four sessions per day. To ensure safety and efficacy, the patient was closely monitored throughout the treatment period. During each session, clinicians assessed for any immediate adverse effects, discomfort, or signs of intolerance. Pain levels, range of motion, and muscle strength were evaluated at regular intervals to track progress and determine the effectiveness of the intervention. Additionally, the patient was encouraged to provide subjective feedback after each session regarding any perceived changes or new symptoms.

**Figure 2:**
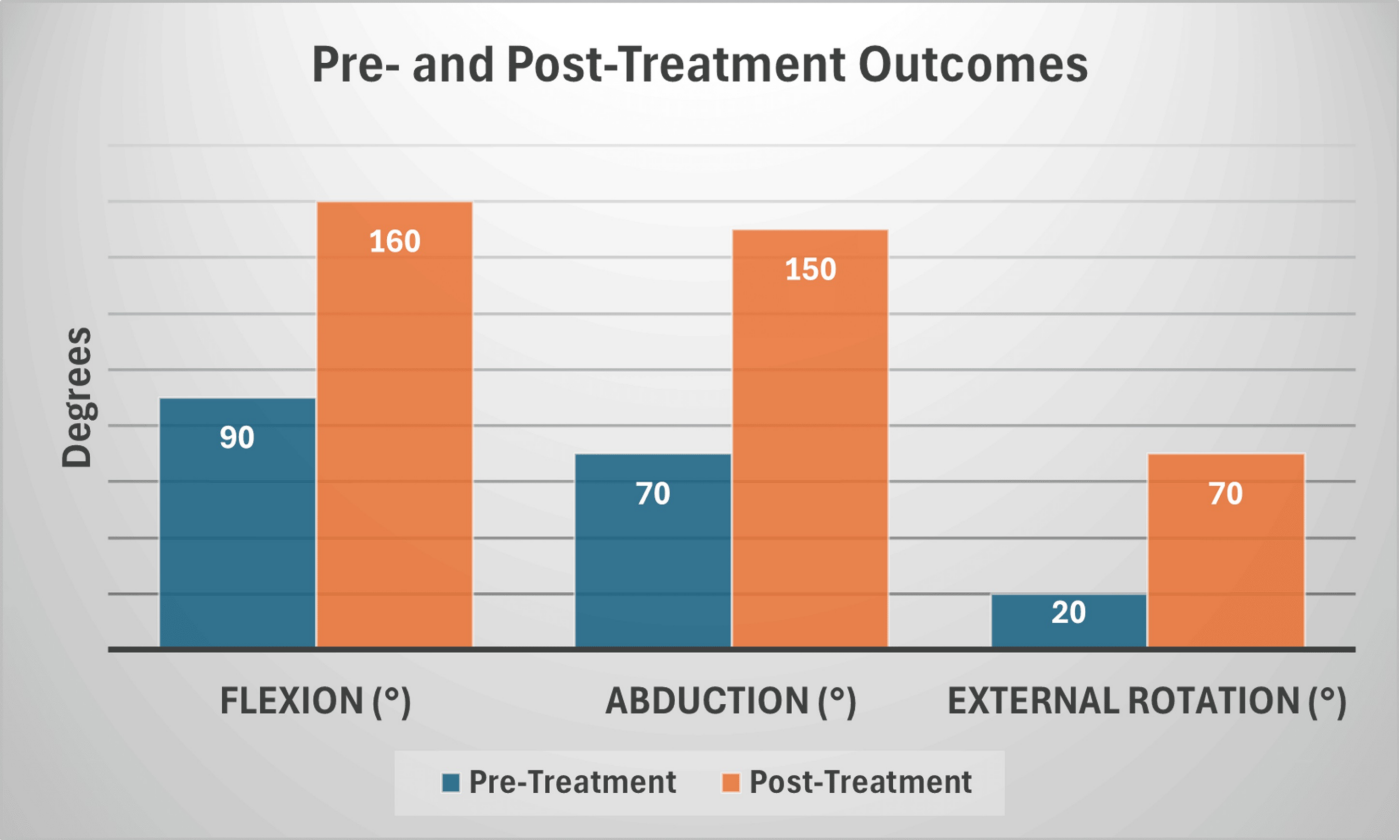
Comparison of pre and post-treatment outcomes. The observed improvements were consistent with the patient’s reported return to normal daily and sports activities without restrictions or residual symptoms, highlighting the comprehensive recovery achieved through the TO-Rpr protocol.

Post-treatment monitoring included follow-up evaluations to confirm the sustained benefits of the protocol. The patient was advised to report any delayed adverse effects or changes in function and to return for clinical assessments if necessary. This comprehensive monitoring approach ensured both the safety of the intervention and the accurate documentation of its outcomes. Substantial improvements were observed following the TO-Rpr treatment cycle. Range of motion (ROM) assessments [12] showed that flexion increased from 90° to 160°, abduction improved from 70° to 150°, and external rotation progressed from 20° to 70° (Figure 2).

Muscle strength evaluations revealed a recovery from 3/5 to 5/5 on the medical research council (MRC) scale [13], while pain assessments using the numeric rating scale (NRS) [14] indicated a reduction from 7/10 to 0/10.

**Figure 3:**
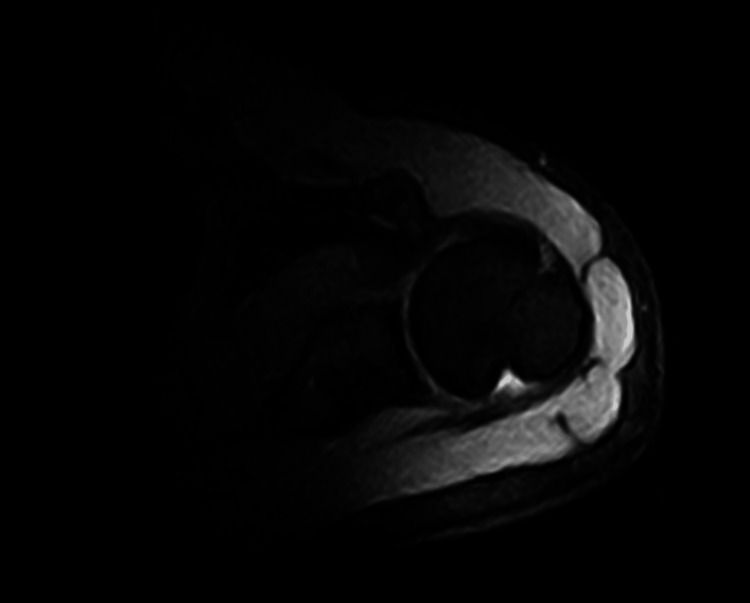
Post treatment magnetic resonance imaging (MRI) of the shoulder. The follow-up scan demonstrates evidence of healing, with reduced inflammation and signs of progressive bone remodeling.

These clinical improvements were corroborated by a follow-up MRI conducted four months after the treatment cycle, which confirmed the resolution of bone marrow edema, normalization of tendinous structures, and a significant reduction in cortical irregularities. No active inflammation or fluid accumulation was detected (Figure 3).

Intermediate improvements were observed during the treatment course. By the third day, the patient reported a noticeable reduction in pain and slight increases in range of motion. These changes were further validated by progressive clinical evaluations conducted during the treatment cycle and at subsequent follow-up visits.

## Discussion

This case highlights the efficacy of the TO-Rpr protocol in addressing the challenges of Hill-Sachs lesions [1,2]. Unlike conventional treatments, TO-Rpr combines bioelectric modulation to target biochemical and biomechanical dysfunctions, promoting tissue repair, reducing inflammation, and restoring functionality through non-invasive means [8-10].

Compared to other non-invasive therapies like platelet-rich plasma (PRP) [15] or extracorporeal shockwave therapy (ESWT) [16], TO-Rpr offers consistent outcomes due to its standardized parameters. PRP and ESWT often depend on operator precision and patient-specific biological responses, whereas TO-Rpr ensures reproducibility and rapid clinical improvements. Additionally, its shorter treatment duration enhances practicality and patient compliance.

In contrast to surgical interventions such as remplissage or bone grafting [17], TO-Rpr avoids risks like infection, prolonged recovery, and post-surgical complications. While surgery effectively stabilizes joints, it often fails to address chronic inflammation and biochemical dysfunctions [18]. TO-Rpr bridges this gap by promoting cellular repair and reducing inflammation without invasive procedures. Its therapeutic effects are supported by its molecular mechanisms. REAC technology modulates bioelectric environments, enhancing cellular communication and reorganizing cytoskeletal structures [10,11,19]. This reduces inflammatory mediators, such as cytokines, and promotes tissue repair. Studies also demonstrate TO-Rpr’s ability to induce positive epigenetic changes, influencing genes involved in cellular proliferation and anti-inflammatory responses [19].

Existing studies validate TO-Rpr’s reparative potential. Provencher et al. emphasized the limitations of traditional approaches, aligning with TO-Rpr’s ability to repair structural damage and reduce inflammation [3]. Funakoshi et al. highlighted gaps in arthroscopic techniques, where TO-Rprs bioelectric modulation offers a clear advantage [2]. Other studies further support the role of REAC technology in promoting cellular repair and regeneration, reinforcing the findings of this case [19,20]. Despite promising results, limitations remain. As a single-case study, the findings lack generalizability.

## Conclusions

The TO-Rpr protocol demonstrated remarkable efficacy in the treatment of a Hill-Sachs lesion, facilitating significant improvements in pain, mobility, and overall functionality. Its reliance on bioelectric modulation offers a patient-centric, non-invasive alternative to traditional treatments. By addressing both structural and symptomatic aspects of joint injuries, TO-RPR represents a promising tool in reparative medicine.

The reproducibility, safety, and tolerability of the protocol further enhance its potential for integration into clinical practice. As advancements in regenerative medicine continue, innovative technologies like REAC may redefine therapeutic approaches for complex musculoskeletal conditions. Future studies should explore its broader applications and establish its role within comprehensive treatment strategies.
